# Reduced Levels of Drp1 Protect against Development of Retinal Vascular Lesions in Diabetic Retinopathy

**DOI:** 10.3390/cells10061379

**Published:** 2021-06-03

**Authors:** Dongjoon Kim, Hiromi Sesaki, Sayon Roy

**Affiliations:** 1 Department of Medicine, Boston University School of Medicine, Boston, MA 02118, USA; djkim@bu.edu; 2 Department of Ophthalmology, Boston University School of Medicine, Boston, MA 02118, USA; 3 Department of Cell Biology, Johns Hopkins University School of Medicine, Baltimore, MD 21205, USA; hsesaki@jhmi.edu

**Keywords:** Drp1, apoptosis, mitochondria, diabetic retinopathy

## Abstract

High glucose (HG)-induced Drp1 overexpression contributes to mitochondrial dysfunction and promotes apoptosis in retinal endothelial cells. However, it is unknown whether inhibiting Drp1 overexpression protects against the development of retinal vascular cell loss in diabetes. To investigate whether reduced Drp1 level is protective against diabetes-induced retinal vascular lesions, four groups of mice: wild type (WT) control mice, streptozotocin (STZ)-induced diabetic mice, Drp1^+/−^ mice, and STZ-induced diabetic Drp1^+/−^ mice were examined after 16 weeks of diabetes. Western Blot analysis indicated a significant increase in Drp1 expression in the diabetic retinas compared to those of WT mice; retinas of diabetic Drp1^+/−^ mice showed reduced Drp1 level compared to those of diabetic mice. A significant increase in the number of acellular capillaries (AC) and pericyte loss (PL) was observed in the retinas of diabetic mice compared to those of the WT control mice. Importantly, a significant decrease in the number of AC and PL was observed in retinas of diabetic Drp1^+/−^ mice compared to those of diabetic mice concomitant with increased expression of pro-apoptotic genes, Bax, cleaved PARP, and increased cleaved caspase-3 activity. Preventing diabetes-induced Drp1 overexpression may have protective effects against the development of vascular lesions, characteristic of diabetic retinopathy.

## 1. Introduction

Diabetic retinopathy is the leading cause of blindness in the working-age population and, unfortunately, there is no cure for this ocular complication [1]. Diabetic retinopathy is characterized by retinal vascular cell loss, a characteristic early stage lesion [2] which manifests as acellular capillaries and pericyte ghosts [3,4]. Increasing evidence indicates that changes in mitochondrial morphology can promote mitochondrial dysfunction and contribute to apoptotic cell death associated with diabetic retinopathy [5,6,7,8,9,10,11,12,13,14,15]. Our recent study has identified mitochondrial fragmentation in vascular cells of retinal capillaries in diabetes [8]. Maintenance of mitochondrial morphology is regulated by fission and fusion events and is integral to mitochondrial functionality. Specifically, imbalance in mitochondrial dynamics through increased mitochondrial fission by dynamin-related protein 1 (Drp1) is known to compromise mitochondrial morphology and lead to mitochondrial dysfunction [16,17]. However, it is unclear whether abnormal changes in Drp1 contribute to the pathophysiology of diabetic retinopathy.

Drp1 is a GTPase that is considered to be a principal regulator of mitochondrial fission [18,19]. It is primarily localized in the cytosol, and upon activation through GTP hydrolysis, it oligomerizes around the mitochondrial outer membrane to initiate fission, mitochondrial fragmentation [20], and ultimately induce apoptosis [21]. Specifically, findings from previous studies overwhelmingly suggest excess fission leads to deleterious effects, including mitochondrial fragmentation and apoptotic cell death [22,23,24]. We have recently reported that retinal endothelial cells grown in 30 mM HG exhibit Drp1 overexpression, and that reducing Drp1 expression protects against HG-induced mitochondrial fragmentation and apoptosis in vitro [10]. However, it is unclear whether decreasing Drp1 upregulation provides beneficial effects against apoptotic cell death.

Drp1 overexpression has been widely reported in HG and diabetic conditions. Podocytes and glomerular mesangial cells grown in HG medium exhibit significant Drp1 upregulation and increased mitochondrial fission, which promote podocyte loss and compromise glomerular function, suggesting that elevated Drp1 plays a role in the pathogenesis of diabetic nephropathy [25,26,27]. In addition, Drp1 overexpression was observed in pancreatic β-islet cells grown in HG medium concomitant with increased mitochondrial fission, cytochrome c release, reduced mitochondrial membrane potential, caspase-3 activation, and reactive oxygen species (ROS) production [28]. However, these reported changes were not evident in pancreatic β-islet cells carrying a dominant negative mutant of Drp1 [28], suggesting that Drp1 plays a critical role in promoting HG-induced apoptosis of pancreatic β-islet cells. Moreover, increased Drp1 levels have been implicated in promoting mitochondrial fragmentation, ROS accumulation, and contributing to apoptotic cell death of endothelial cells in models of atherosclerosis and diabetic cardiomyopathy [29,30]. Elevated Drp1 expression and increased mitochondrial fission were also observed in the dorsal root ganglion and hippocampus of diabetic animals, suggesting that Drp1 overexpression may contribute to the pathogenesis of diabetic neuropathy [31,32]. Taken together, these findings indicate a critical role for Drp1 in promoting mitochondrial fragmentation and apoptosis under HG and diabetic conditions.

To determine whether increased levels of Drp1 contribute to the development of apoptotic death of vascular cells in the diabetic retina, in the present study, we induced diabetes in the Drp1^+/−^ mouse and investigated whether reduced levels of Drp1 in these mice were protective against the development of acellular capillaries and pericyte loss. Specifically, proapoptotic genes Bax, cleaved PARP, and caspase-3 activity were examined in addition to TUNEL assays, which were performed to identify vascular cells undergoing apoptosis in retinal capillaries.

## 2. Materials and Methods

### 2.1. Animals

Studies involving animals were carried out following the guidelines of the ARVO Statement for the Use of Animals in Ophthalmic and Vision Research and approved by the IACUC Committee of Boston University (PROTO201800411; approved on March 9th, 2021). Male and female mice were used in the present study to address any sex-related differences. A total of 12 male and 12 female WT C57BL/6J mice (The Jackson laboratory, Bar Harbor, Maine), as well as 12 male and 12 female Drp1^+/−^ mice bred into the C57/BL6J strain background provided by Dr. Hiromi Sesaki [33] were used to conduct experiments. A detailed methodology on how Drp1^+/−^ mouse model was generated can be referred to in a previous study [33]. Polymerase chain reaction (PCR) was performed using tail tip DNA of animals to verify their genotype. PCR was carried out with primer sequences as follows: Primer 1, 5′-ACCAAAGTAAGGAATAGCTGTTG-3′; Primer 2, 5′-GAGTACCTAAAGTGGACAAGAGGTCC-3′; Primer 3, 5′-CACTGAGAGCTCTATATGTAGGC-3′). Drp1^+/−^ allele is represented as a 539-bp fragment amplified by primers 1 and 2. Drp1^+/+^ allele is represented as a 315-bp fragment amplified by primers 2 and 3. In the present study, Drp1^−/−^ mice were not used as this genotype is embryonically lethal [33,34,35].

12 WT mice and 12 Drp1^+/−^ mice were randomly assigned to receive 5 consecutive STZ injections intraperitoneally to induce diabetes at a concentration of 40 mg/kg body weight. Additionally, 12 WT mice and 12 Drp1^+/−^ mice were randomly assigned to receive 5 consecutive citrate buffer injections intraperitoneally as vehicle, representing non-diabetic control groups. To verify the diabetes status in the animals, blood and urine glucose levels were measured 3 days post-STZ injection. Routine blood glucose assessment was performed 3 times per week. Depending on the hyperglycemic status, NPH insulin injections were administered to achieve a level of ~350 mg/dL. A total of 16 weeks after the onset of diabetes, animals were sacrificed, blood was collected from each animal and blood glucose and HbA1c levels were measured. Following sacrifice, retinas from each animal were isolated, and total protein extracted from all samples.

### 2.2. Immunostaining of Drp1 in Retinal Capillary Networks

To study the expression and distribution of Drp1 in the retinal capillary networks, retinal trypsin digestion (RTD) preparations [36] were subjected to immunostaining with Drp1 antibody. The RTD preparations were washed several times with 1× PBS and subjected briefly to ice-cold methanol, followed by additional PBS washes. Then, the RTDs were exposed to a 2% BSA solution diluted in 1× PBS for 15 min at room temperature to block non-specific antibody binding. Following blocking, the RTDs were subjected to a primary antibody solution containing mouse monoclonal Drp1 antibody (1:200 in 2% BSA-PBS solution, Catalog #sc-271583, Santa Cruz Biotechnology, Dallas, TX, USA) and incubated overnight at 4 °C in a moist chamber. After the overnight incubation, the RTDs were washed in PBS and incubated at room temperature with FITC-conjugated rabbit anti–mouse IgG secondary antibody (1:100 in 2% BSA-PBS Solution, Jackson for 1 h). After three PBS washes, RTDs were counterstained with DAPI, and mounted in SlowFade Diamond Antifade Mountant reagent (SlowFade Diamond; Molecular Probes, Eugene, OR, USA). Digital images were captured, and relative Drp1 immunofluorescence was quantified using the NIH Image J software from 10 random representative fields from each RTD.

### 2.3. Western Blot Analysis

WB analysis was carried out as described [10]. Briefly, total protein was isolated from retinas of experimental animals using a lysis buffer solution containing 10 mmol/L Tris, pH 7.5 (Sigma, Temecula, CA, USA), 1 mmol/L EDTA, and 0.1% Triton X-100 (Sigma). Bicinchoninic acid assay (Pierce Chemical, Rockford, IL, USA) was used to obtain protein concentrations of the retinal lysates, which were then subjected to WB analysis for Drp1, Bax, and PARP activation. Equal amount of protein (20 μg) of retinal lysates was loaded into each lane in a 10% SDS-polyacrylamide gel and electrophoresed, followed by semi-dry transfer [37] using a PVDF membrane (Millipore, Billerica, MA, USA). Following transfer, the membrane was exposed to a blocking solution containing 5% non-fat dry milk for 1 h and subsequently incubated overnight at 4 °C with mouse monoclonal Drp1 antibody (1:1000, Catalog #sc-271583, Santa Cruz Biotechnology), rabbit Bax antibody (1:1000, Catalog #2772, Cell Signaling, Danvers, MA, USA), or PARP antibody (1:500, Catalog #9542, Cell Signaling) in a solution comprised of 5% bovine serum albumin dissolved in 0.1% Tween-20 (TTBS). The following day, the membrane was subjected to incubation with a secondary antibody solution (anti-rabbit IgG, AP-conjugated antibody (1:3000, Catalog #7054, Cell Signaling) or anti-mouse IgG, AP-conjugated antibody (1:3000, Catalog #7056, Cell Signaling)) for 1 h in room temperature. The membrane was then exposed to a chemiluminescent substrate (Bio-Rad, Hercules, CA, USA) and chemiluminescence signals were captured using a digital imager (Fujifilm LAS-4000). The membrane underwent Ponceau-S staining after transfer or was re-probed with β-actin antibody (1:1000, Catalog #4967, Cell Signaling) to confirm equal loading. NIH Image J software was used to conduct densitometric analysis of the chemiluminescent signal non-saturating exposures.

### 2.4. Assessment of Caspase-3 Activity

To evaluate caspase-3 activity in retinas of diabetic animals and Drp1^+/−^ animals, fluorometric analysis was carried out using a commercially available caspase-3 assay kit (Abcam, Cambridge, UK; Catalog #ab39383). Lysates from retinal tissues were isolated using the kit’s proprietary lysis buffer, incubated on ice for 10 min, and homogenized. Following BCA assay of the retinal tissue lysates, 20 μg of protein from each sample was used to perform the fluorometric evaluation of caspase-3 activity. The retinal lysates were mixed with reaction buffer containing DTT (10 mM final concentration) and Acetyl-Asp-Glu-Val-Asp-7-amino-4 trifluoromethylcoumarin (DEVD-AFC) (50 µM final concentration), a fluorogenic substrate specific to caspase-3. The reaction mixture representing each sample was transferred to corresponding wells in a 96-well plate, incubated at 37 °C for 2 h, and subjected to fluorescent excitation and emission at 400 nm and 505 nm, respectively. Specifically, cleavage of the DEVD-AFC substrate is carried out by activated caspase-3 resulting in the formation of free AFC molecules, which can be detected at 400 nm excitation and 505 nm emission [38]. Therefore, relative difference in DEVD-AFC cleavage between experimental groups was used to analyze caspase-3 activity.

### 2.5. Retinal Trypsin Digestion and Assessment of Acellular Capillaries and Pericyte Loss

After animals were sacrificed, eyes were enucleated and placed in 10% formalin, and retinas isolated and exposed to 0.5 M glycine for 24 h. To isolate retinal capillaries, RTD was performed as described [36]. Briefly, retinas were subjected to 3% trypsin, glia removed through tapping with a single hair brush, and mounted on a silane-coated slide. RTDs were stained with periodic acid-Schiff and hematoxylin as described [39]. Using a digital camera attached to a microscope (Nikon Eclipse; TE2000-S, Nikon, Tokyo, Japan), ten representative fields were imaged assessed for AC and PL. Vessels without endothelial cells and pericytes represented ACs. PL was determined by counting pericyte ghosts, which appear as “empty shells” representing dead pericytes.

### 2.6. Terminal dUTP Nick-End Labeling Assay

To detect cells undergoing apoptosis in retinal capillaries, terminal deoxynucleotidyl transferase-mediated uridine 5′-triphosphate-biotin nick end labeling (TUNEL) assay was performed using a kit (ApopTag Fluorescein In Situ Apoptosis Detection; Millipore Sigma) as described previously [39]. Briefly, RTDs were fixed in paraformaldehyde, permeabilized in a pre-cooled mixture of a 2:1 ratio of ethanol/acetic acid, washed in PBS, exposed to equilibration buffer, and incubated for 1 h with deoxyribonucleotidyl transferase (TdT) enzyme in a moist chamber at 37 °C. Following incubation, RTDs were exposed to anti-digoxigenin peroxidase, washed in PBS, counterstained with DAPI, and mounted using anti-fade reagent (SlowFade Diamond Antifade, Cat#S36963; Invitrogen, Carlsbad, CA, USA). At least five images representing random fields of the RTD slide were captured using a digital microscope (Nikon Eclipse; TE2000-S) and TUNEL-positive cells per total number of cells per field were analyzed.

### 2.7. Statistical Analysis

Data are shown as mean ± standard deviation. Values representing experimental groups are shown as percentages of the control. The normalized values were subjected to Student’s *t*-test for comparisons between two groups, or one-way ANOVA followed by Bonferroni’s post-hoc test for comparisons between multiple groups. Statistical significance was considered at *p* < 0.05.

## 3. Results

### 3.1. Drp1^+/−^ Animal Model

**Figure 1:**
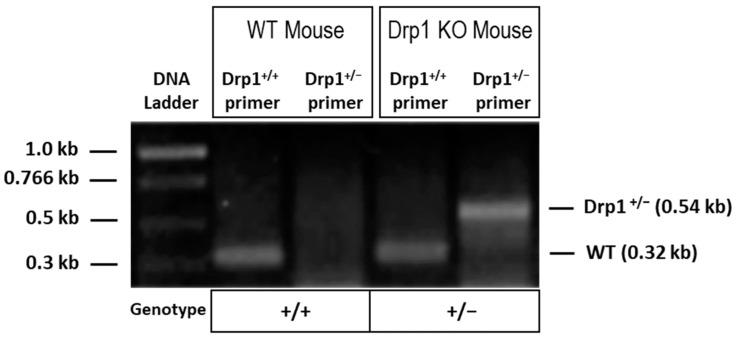
PCR analysis using mice tail tip DNA indicating genotypes of Drp1 heterozygous knockout (^+/−^) and wild-type (WT) mice. The wild-type Drp1 allele (Drp1^+/+^) is represented by a band at 0.32 kb, whereas the disrupted allele (Drp1^+/−^) shows a band at 0.54 kb.

Genotypes of animals used in the present study were confirmed through PCR analysis using DNA derived from the animals’ tail tips. PCR data was used to confirm that wild-type (WT) mice (Drp1^+/+^) exhibited a band as expected at 0.54 kb whereas Drp1 heterozygous knockout (Drp1^+/−^) mice exhibited a band as expected at 0.32 kb (Figure 1).

### 3.2. Effect of Diabetes on Drp1 Expression and Distribution in Retinal Capillary Networks

**Figure 2:**
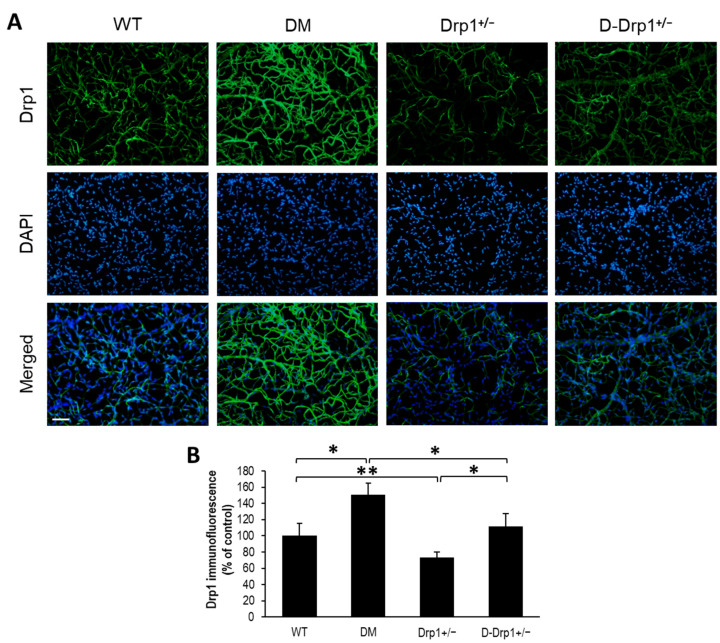
Drp1^+/−^ mice exhibit reduced Drp1 immunofluorescence in the retinal capillary network. (**A**) Representative images of Drp1 immunofluorescence (green) and DAPI (blue) in retinal capillaries of wild-type (WT), diabetic (DM), Drp1^+/−^, and diabetic Drp1^+/−^ (D-Drp1^+/−^) mice. Scale bar = 100 μm. (**B**) Graphical illustration of cumulative data shows decreased Drp1 immunofluorescence in retinal capillaries of Drp1^+/−^ mice compared to that of WT mice. * *p* < 0.01, *n* = 6; ** *p* < 0.05, *n* = 6.

To determine whether the distribution of Drp1 is altered in retinal capillary networks of diabetic animals and Drp1^+/−^ animals, Drp1 immunostaining was performed in RTDs from each experimental group. Interestingly, Drp1 immunostaining was significantly increased in RTDs of diabetic mice compared to that of non-diabetic WT mice (151 ± 14% of WT vs. 100 ± 15% of WT, *p* < 0.01; *n* = 6; Figure 2A,B). As expected, Drp1 immunostaining was significantly decreased in RTDs of Drp1^+/−^ mice compared to that of non-diabetic WT mice (73 ± 7% of WT vs. 100 ± 15% of WT, *p* < 0.05; *n* = 6; Figure 2A,B). Drp1 immunostaining was significantly decreased in RTDs of diabetic Drp1^+/−^ mice compared to that of diabetic mice (111 ± 16% of WT vs. 151 ± 14% of WT, *p* < 0.01; *n* = 6; Figure 2A,B), and significantly increased compared to non-diabetic Drp1^+/−^ mice (111 ± 16% of WT vs. 73 ± 7% of WT, *p* < 0.01; *n* = 6; Figure 2A,B).

### 3.3. Normalization of Drp1 Expression in Retinas of Diabetic Drp1^+/−^ Mice

**Figure 3:**
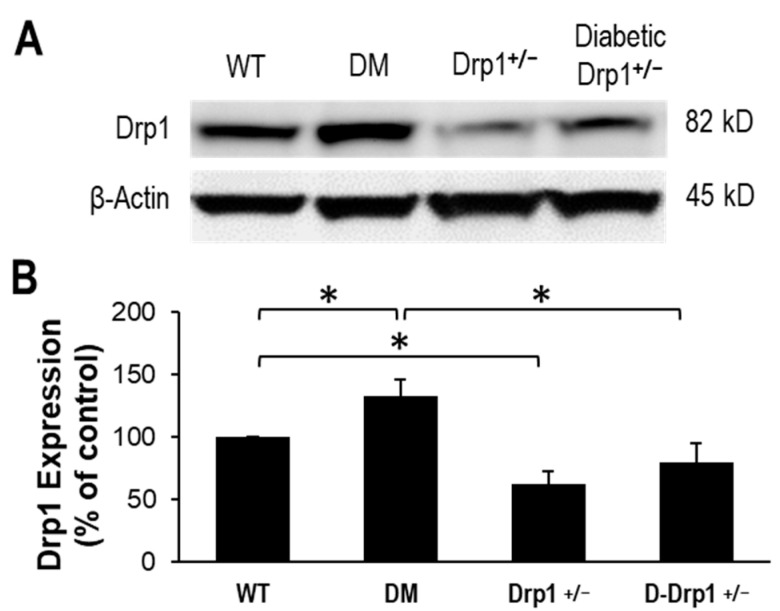
Drp1 expression is normalized in diabetic Drp1 ^+/−^ mouse retinas. (**A**) Representative WB image shows Drp1 protein levels in the retinas of WT, diabetic (DM), Drp1^+/−^, and diabetic Drp1^+/−^ (D-Drp1^+/−^) mice. (**B**) Graphical illustration of cumulative data shows diabetes significantly upregulates Drp1 expression and that Drp1 expression is normalized in retinas of D-Drp1^+/−^ mice. Data are expressed as mean ± SD. * *p* < 0.01, *n* = 12.

Data from Western blot analysis showed that Drp1 expression level is significantly upregulated in diabetic mouse retinas compared to that of WT mouse retinas (133 ± 13% of WT, *p* < 0.01; *n* = 12; Figure 3A,B). As expected, retinas of Drp1^+/−^ exhibited a significant decrease in retinal Drp1 expression compared to that of WT mice (63 ± 10% of WT, *p* < 0.01; *n* = 6; Figure 3A,B). In parallel, Drp1 expression was brought to near normal levels in retinas of diabetic Drp1^+/−^ mice (80 ± 16% of WT, *p* < 0.01; *n* = 12; Figure 3A,B) compared to diabetic mice.

### 3.4. Diabetes-Induced Drp1 Upregulation Promotes Apoptosis

**Figure 4:**
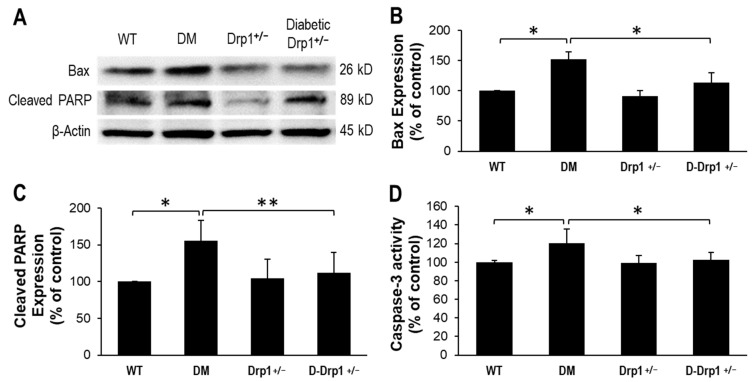
Reduced Drp1 expression lowers Bax activity in diabetic Drp1^+/−^ mouse retinas. (**A**) Representative WB image shows Bax and cleaved PARP expression in the retinas of WT, diabetic (DM), Drp1^+/−^, and diabetic Drp1^+/−^ (D-Drp1^+/−^) mice. Graphical illustrations of cumulative data suggest reduced Drp1 levels in D-Drp1^+/−^ mice is protective against diabetes-induced increase in (**B**) Bax levels, (**C**) PARP cleavage and (**D**) caspase-3 activity. Data are expressed as mean ± SD. * *p* < 0.01, *n* = 12; ** *p* < 0.05, *n* = 12.

To assess whether reduced Drp1 level is protective against diabetes-induced pro-apoptotic genes, expression levels of Bax and cleaved PARP, as well as caspase-3 activity were monitored in retinal tissues. In retinas of diabetic mice, gene expression levels of pro-apoptotic Bax and cleaved PARP were significantly increased (Bax: 152 ± 23% of WT, *p* < 0.01; *n* = 12; Figure 4A,B; Cleaved PARP: 156 ± 27% of WT, *p* < 0.01; *n* = 12; Figure 4A,C) concomitant with increased caspase-3 activity (120 ± 16% of WT, *p* < 0.01; *n* = 12; Figure 4D) compared to those of non-diabetic WT mice. Interestingly, reduced Drp1 level in retinas of diabetic Drp1^+/−^ mice showed a decrease in diabetes-induced Bax expression (113 ± 19% of WT, *p* < 0.01; *n* = 12; Figure 4A,B), PARP cleavage (112 ± 28% of WT, *p* < 0.01; *n* = 12; Figure 4A,C), and caspase-3 activation (102 ± 8% of WT, *p* < 0.01; *n* = 12; Figure 4D).

### 3.5. Drp1 Downregulation Inhibits Vascular Cell Apoptosis in the Diabetic Retina

**Figure 5:**
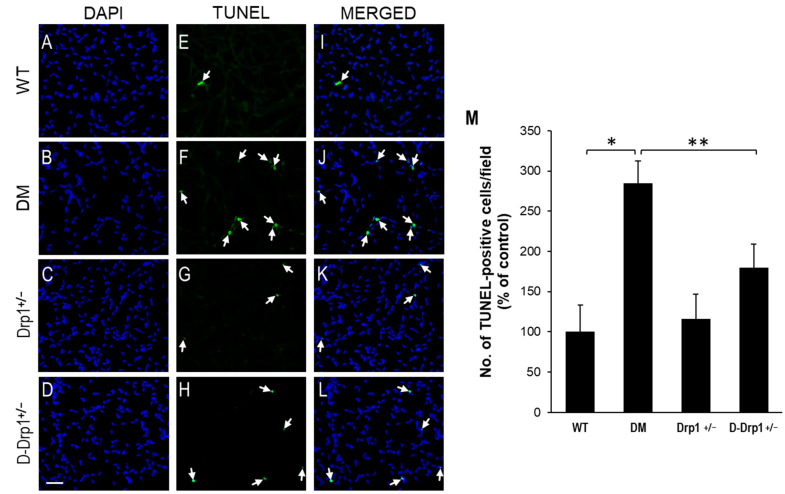
Reduced Drp1 level protects against diabetes-induced apoptosis of vascular cells in retinal capillary networks. Representative images of capillary networks showing DAPI-stained cells in the (**A**) WT, (**B**) diabetic (DM), (**C**) Drp1^+/−^, and (**D**) diabetic Drp1^+/−^ (D-Drp1^+/−^) mice. (**E**–**H**) Corresponding images of TUNEL-positive cells (arrows) in the retinal capillary networks, respectively. (**I**–**L**) Merged images showing DAPI-stained cells superimposed with TUNEL-positive cells. Scale bar = 100 μm. (**M)** Graph of cumulative data showing that retinal capillary networks of diabetic mice exhibited an increase in number of TUNEL-positive cells compared to that of WT mice, while retinal capillary networks of D-Drp1^+/−^ mice showed reduced number of TUNEL-positive cells compared to that of diabetic mice. Data are presented as mean ± SD. * *p* < 0.01, *n* = 6; ** *p* < 0.05, *n* = 6.

To assess whether downregulating Drp1 expression protects apoptotic cell death in retinal vascular cells, TUNEL assay was performed in RTDs from each experimental group. Data indicate that there is a significant increase in the number of TUNEL-positive cells in RTDs of diabetic mice compared to that of non-diabetic WT mice (284 ± 28% of WT vs. 100 ± 33% of WT, *p* < 0.01; *n* = 6; Figure 5A–M). Importantly, when diabetes-induced Drp1 overexpression was brought to near normal levels, the number of TUNEL-positive cells was significantly reduced in RTDs of diabetic Drp1^+/−^ mice compared to that of diabetic mice (180 ± 29% of WT vs. 284 ± 28% of WT, *p* < 0.05; *n* = 6; Figure 5A–M).

### 3.6. Reduced Drp1 Level Is Protective against Diabetes-Induced Development of AC and PL

**Figure 6:**
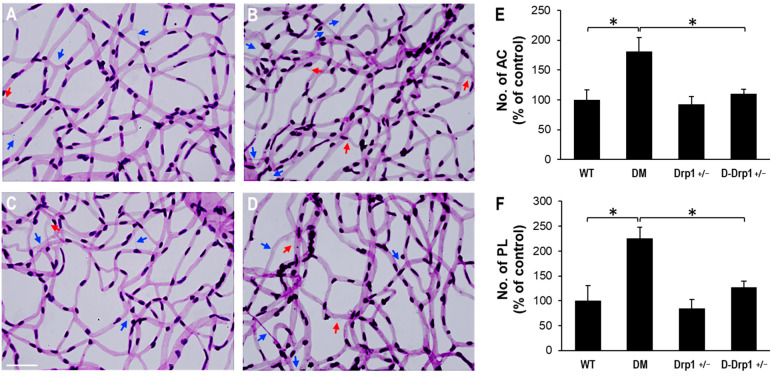
Effects of diabetes or decreased Drp1 level on AC and PL development in mouse retinas. (**A**–**D**) Representative images of retinal trypsin digestion of (**A**) WT, (**B**) DM, (**C**) Drp1^+/−^, and D-Drp1^+/−^ mice show the number of AC (blue arrows) and PL (red arrows) is increased in the diabetic mouse retina compared to that of control mouse retina. Importantly, the number of AC and PL is decreased in the D-Drp1^+/−^ mouse retina compared to that of diabetic mouse retina. Scale bar = 100 μm. Graphical illustrations of cumulative data show that reduced DRP1 levels in the D-Drp1^+/−^ mouse retina exhibited a protective effect against the development of (**E**) AC and (**F**) PL. Data are expressed as mean ± SD. * *p* < 0.01, *n* = 12.

To evaluate the effects of diabetes and reduced Drp1 levels in the development of AC and PL, retinal trypsin digestion was carried out and the numbers of AC and PL between the experimental groups were analyzed. RTD data indicate a significant increase in the numbers of AC and PL in retinal capillary networks of diabetic mice compared to those of non-diabetic WT mice (AC: 181 ± 23% of WT vs. 100 ± 17% of WT, *p* < 0.01; *n* = 12; Figure 6A–F, PL: 225 ± 23% of WT vs. 100 ± 31% of WT, *p* < 0.01; *n* = 12; Figure 6A–F). Importantly, when Drp1 levels were reduced in diabetic Drp1^+/−^ mice, the numbers of AC and PL significantly decreased compared to those of diabetic mice (AC: 111 ± 7% of WT vs. 181 ± 23% of WT, *p* < 0.01; *n* = 12; Figure 6A–F, PL: 128 ± 11% of WT vs. 225 ± 23% of WT, *p* < 0.01; *n* = 12; Figure 6A–F). RTDs of Drp1^+/−^ mice exhibited no significant difference in the numbers of AC and PL compared to those in non-diabetic WT mice (AC: 93 ± 13% of WT vs. 100 ± 17% of WT, *p* > 0.05; *n* = 12; Figure 6A–F, PL: 85 ± 17% of WT vs. 100 ± 31% of WT, *p* > 0.05; *n* = 12; Figure 6A–F).

## 4. Discussion

Present study provides novel evidence that Drp1 expression is significantly increased in retinas of diabetic mice, and that reduced levels of Drp1 provide beneficial effects in preventing apoptotic death of retinal vascular cells and subsequent development of acellular capillaries and pericyte loss characteristic of diabetic retinopathy. Of note, no significant adverse effects were observed in the retina and other tissues of Drp1^+/−^ animals exhibiting ~40% reduction in Drp1 levels. Similarly, data from the present study showed no significant sex-related differences in neither WT or Drp1^+/−^ animals. These findings indicate that increased Drp1 level is closely associated with the development of retinal vascular lesions, and that reducing it could prevent apoptotic vascular cell death associated with diabetic retinopathy.

Mitochondrial morphology and functionality are intrinsically linked [7]. Breakdown in mitochondrial morphology is known to compromise mitochondrial function [6,9,10]. Excess mitochondrial fission resulting from increased Drp1 levels can disturb the delicate balance between mitochondrial fission and fusion events, promote mitochondrial fragmentation and ultimately compromise mitochondrial functionality [16,17,40,41,42,43,44,45]. Additionally, Drp1-driven mitochondrial fragmentation can undermine mitochondrial respiration, alter calcium storage, and lead to increased ROS production [40,43]. Another study reported that changes due to increased Drp1-mediated mitochondrial fragmentation impair metabolic functions, which compromises mitochondrial homeostasis in neuroinflammation [44]. Importantly, inhibition of Drp1-induced mitochondrial fragmentation led to improvements in mitochondrial functionality as evidenced by restoration of mitochondrial membrane potential and mitochondrial respiration [10,42]. Taken together, these reports provide further evidence that excess Drp1-driven mitochondrial fragmentation contributes to impaired mitochondrial functionality in diabetic condition.

While studies have established that participation of Drp1 is essential in regulating mitochondrial fission, mechanisms underlying Drp1 upregulation and its role in inducing apoptosis remain unclear. Increased Drp1 activation triggers apoptosis by promoting translocation of Bax to mitochondria and ultimately activating caspase-3 signaling [46]. In addition, Drp1-driven mitochondrial fission contributes to mitochondrial fragmentation by promoting outer mitochondrial membrane permeabilization, hindering ATP production, and triggering release of pro-apoptotic factors [41] as well as facilitating mitochondrial division leading to oligomerization of Bax and cytochrome c release [47,48], ultimately inducing apoptosis. Interestingly, Drp1^−/−^ cells are protected against apoptosis [49] and Drp1 inhibition reduced cleavage of caspase-3 and PARP in hepatocytes [50], suggesting that targeting Drp1 may be protective against apoptosis. Further studies are necessary to better understand the diverse mechanisms implicated in excess Drp1-mediated apoptosis.

Growing evidence shows that blocking Drp1 overexpression could be effective in preventing mitochondrial fission and protecting against apoptotic cell death [24,51,52,53]. Inhibition of Drp1-mediated mitochondrial fission reduced ER stress response in fibroblasts, which in turn reduced cellular stress and improved cell survival [54]. Importantly, selective inhibition of Drp1 using Mdivi-1 hindered Drp1 self-assembly, which effectively blocked Bax-dependent cytochrome c release and mitochondrial outer membrane permeabilization, ultimately preventing apoptosis [51]. Moreover, Drp1 downregulation inhibited mitochondrial fragmentation and prevented cytochrome c release and caspase activation [24,53]. Maintenance of Drp1 levels preserves mitochondrial cristae ultrastructure and prevents cytochrome c release and the downstream apoptotic signaling cascade [52]. Our previous report [10] as well as studies from other investigators [29,53,54] support our current finding that reducing Drp1 overexpression could be beneficial against diabetes-induced apoptosis in retinal vascular cells, and that targeting Drp1 overexpression could be a useful strategy against the development of retinal vascular lesions associated with diabetic retinopathy.

## Data Availability

Data presented in the article are available by request to corresponding author, Sayon Roy (sayon@bu.edu).
